# Testing adults by questionnaire for social and communication disorders, including autism spectrum disorders, in an adult mental health service population

**DOI:** 10.1002/mpr.1814

**Published:** 2020-01-10

**Authors:** Traolach Brugha, Freya Tyrer, Andrew Leaver, Samantha Lewis, Sarah Seaton, Zoe Morgan, Samuel Tromans, Kobus van Rensburg

**Affiliations:** ^1^ Department of Health Sciences University of Leicester Leicester UK; ^2^ Leicestershire Partnership NHS Trust Leicester UK; ^3^ Northamptonshire Healthcare NHS Foundation Trust Northampton UK

**Keywords:** adults, autism, epidemiology, psychological tests

## Abstract

**Objectives:**

Autism is difficult to identify in adults due to lack of validated self‐report questionnaires. We compared the effectiveness of the autism‐spectrum quotient (AQ) and the Ritvo autism–Asperger's diagnostic scale‐revised (RAADS‐R) questionnaires in adult mental health services in two English counties.

**Methods:**

A subsample of adults who completed the AQ and RAADS‐R were invited to take part in an autism diagnostic observation schedule (ADOS Module 4) assessment with probability of selection weighted by scores on the questionnaires.

**Results:**

There were 364 men and 374 women who consented to take part. Recorded diagnoses were most commonly mood disorders (44%) and mental and behavioural disorders due to alcohol/substance misuse (19%), and 4.8% (95% CI [2.9, 7.5]) were identified with autism (ADOS Module 4 10+). One had a pre‐existing diagnosis of autism; five (26%) had borderline personality disorders (all female) and three (17%) had mood disorders. The AQ and RAADS‐R had fair test accuracy (area under receiver operating characteristic [ROC] curve 0.77 and 0.79, respectively). AQ sensitivity was 0.79 (95% CI [0.54, 0.94]) and specificity was 0.77 (95% CI [0.65, 0.86]); RAADS‐R sensitivity was 0.75 (95% CI [0.48, 0.93]) and specificity was 0.71 (95% CI [0.60, 0.81]).

**Conclusions:**

The AQ and RAADS‐R can guide decisions to refer adults in mental health services to autism diagnostic services.

## 1 INTRODUCTION

Social and communication disorders, including autism spectrum disorders (ASDs), contribute to substantial societal burden (Knapp et al., 2009) and can be challenging for adults and carers involved in providing and accessing support (Commission for Social Care Inspection, 2008, Department of Health, 2006). Improving recognition and diagnosis of ASD became a cross‐government policy in England in 2009 with the passing of the Autism Act, the Think Autism Strategy (Health, 2014), and statutory guidance to the National Health Service and local authorities in 2015 (Health, 2015). Many high‐functioning adults with ASD have not been diagnosed (National Audit Office, 2009; Brugha et al., 2011). Such recognition can be valuable because a diagnosis opens up a range of autism services, such as social groups, support to live independently, and support with finding and remaining in employment. With the right support, many adults with ASD can live and work independently, and lead fulfilling and rewarding lives.

Most adults living with ASD do not have intellectual disability, though ASD is more prevalent within this group (Brugha et al., 2016). It is difficult to identify ASD in adults who have not been diagnosed in childhood because existing diagnostic instruments ideally require input from a parent who is often not available. Self‐completion questionnaire tests have the potential to be useful, but have not been validated in representative study populations (Wigham et al., 2018), and evaluations to date have shown there is very limited evidence to support their use in the assessment and diagnosis of ASD in adults. Mental health settings are a particularly important population for study because ASD is more common in these settings (Nylander & Gillberg, 2001), has the potential to be “masked” by other conditions (Kopp & Gillberg, 1997; Rastam, 2008; Rydén, Rydén, & Hetta, 2008) or can be erroneously diagnosed because of symptom overlap with other conditions.

With the above in mind, this study focused on validating the two most common adult self‐completion ASD questionnaires: the autism‐spectrum quotient (AQ; Baron‐Cohen, Wheelwright, Skinner, Martin, & Clubley, 2001) and the Ritvo autism–Asperger's diagnostic scale‐revised (RAADS‐R; Ritvo et al., 2011) for the first time in representative mental health settings with a view to facilitating mental health professionals' referrals to specialist autism services. The study forms part of a wider programme to assess acceptability, content validity, criterion‐related validity, and reliability of the AQ and RAADS‐R. This article presents findings from the latter two components (criterion‐related validity and reliability).

## 2 METHOD

The target population was drawn from adult mental health service users of psychiatric inpatient units, outpatient units, and community mental health services served by the relevant trusts in Leicestershire and Northamptonshire, England. Approximately 1,000 service users are seen in these settings per month. Secure and elderly psychiatric units were excluded from the study owing to the capacity to consent issues. In total, 46 potential mental health sites were identified and their clinical leads invited to take part in the study; 32 (70%) agreed to help with recruitment.

Between August 2011 and December 2012, adult (aged 18+) mental health service users from the participating mental health sites were approached by members of the research team and specially trained research volunteers to take part in the study. All adults, regardless of recorded diagnosis, were deemed eligible to be approached with a few exceptions, namely, adults who could not speak English, had intellectual disability or lacked capacity to consent (normally under advice from the acting clinician), or whose contact was with one specialist service for eating disorders. Participants were asked to complete the AQ and RAADS‐R, with the option of completing them in the presence of the researcher or taking the questionnaires home to complete.

As well as basic demographic information, recorded primary psychiatric diagnoses within 2 years prior to questionnaire completion and diagnoses of ASD or attention deficit hyperactivity disorder within 5 years prior to questionnaire completion were collected for all consenting adults. This was achieved via a combination of reviewing their clinical letters as well as accessing electronic healthcare records. In the case of service users recruited within the community setting, all were approached prior to their interview with the diagnostic clinician and thus any new diagnoses as a result of this subsequent clinical interview were not recorded.

Adults were selected for a second home interview with the autism diagnostic observation schedule (ADOS Module 4; Lord et al., 2000), with a greater likelihood of selection, the higher their score in the AQ and/or RAADS‐R questionnaires (Table 1). In September 2012, when recruitment was slowing, the probabilities of selection were changed to increase the number of service users selected for a second interview.

**Table 1:** Probability of selection for second interview by total scores in the autism–spectrum quotient (AQ) and Ritvo autism–Asperger's diagnostic scale‐revised (RAADS‐R)

		Pre September 2012		Post September 2012		Total
		Probability	*N*	Probability	*N*	
Total AQ score	0–19	0.1	126	0.3	31	157
	20–24	0.2	112	0.6	18	130
	25–29	0.3	62	0.9	11	72
	30–39	0.6	69	1.0	18	86
	40+	1.0	9	1.0	1	10
	Total	—	378	—	79	457
Total RAADS‐R score	0–49	0.1	92	0.3	24	116
	50–79	0.2	79	0.6	10	89
	80–129	0.3	126	0.9	27	153
	130–179	0.6	60	1.0	10	70
	180+	1.0	8	1.0	2	10
	Total	—	365	—	73	438

To assess test–retest reliability, a random sample of participants was contacted within 1–3 months of completing their first questionnaire to complete the questionnaires again.

### 2.1 Instruments

The AQ (Baron‐Cohen et al., 2001) is a 50‐item self‐completion questionnaire that identifies autistic traits in adults with typical (normal) IQ. Responses are given in four ordinal responses, dichotomised for scoring, for whether a respondent agrees or does not agree with the given statements. The developers of the AQ initially assessed its effectiveness, using a case–control design, in university students and winners of a UK mathematical Olympiad who were compared with adults with high‐functioning autism/ASD recruited through the National Autistic Society, clinics, and advertisements, finding that the AQ discriminated well between participants with and without ASD (Baron‐Cohen et al., 2001), with a lower cutoff threshold recommended for screening in clinical practice (Woodbury‐Smith, Robinson, Wheelwright, & Baron‐Cohen, 2005).

The RAADS‐R is an 80‐item questionnaire completed by the service user with support from a clinician (Ritvo et al., 2011). Four response options are offered, measuring whether the respondent judges the given statement to be true now and/or in childhood or never true. The developers of the RAADS‐R evaluated its efficacy, using a case–control design, in three specially selected groups of adults in centres in the United States and Europe: adults with confirmed autism or Asperger syndrome (*n* = 201), adults with other mental disorders who were not autistic (*n* = 302), and adults with no previous mental health disorders (*n* = 276). The RAADS‐R discriminated well between the groups (100% specificity; 97% sensitivity; Ritvo et al., 2011), but the mean RAADS‐R scores in the nine research centres were significantly different, and there was potential for clinician bias in supporting the participant to complete the questionnaires.

The reference standard used to assess criterion‐related validity was the ADOS Module 4 (Lord et al., 2000) already validated in the adult general population in England in comparison with two autism standardised assessments, the Diagnostic Interview for Social and Communication Disorders (DISCO) and Autism Diagnostic Interview ‐ Revised (ADI‐R) (Brugha et al., 2012). The ADOS is a widely used instrument for assessing behaviours described in autism, developed by Catherine Lord et al (Lord, Rutter, Dilavore, & Risi, 2002). The assessment consists of a series of structured and semi‐structured tasks that involve social interaction between the interviewer and individual. Module 4 is designed for adults with fluent verbal ability. Interviewers completed extensive training and reliability assessment as described previously (Brugha et al., 2011).

### 2.2 Statistical methods

For the purposes of this study, we allowed up to three missing values in the questionnaires: Missing values were then imputed as the average (mean) of the other items in the respective questionnaire. Population demographics, psychiatric diagnoses, and the distribution of AQ and RAADS‐R scores were described and tested for normality. Internal consistency of items on the questionnaires was assessed using Cronbach's alpha (Cronbach, 1951). Spearman's correlations between AQ and RAADS‐R scores with the ADOS Module 4 were calculated. Receiver operating characteristic (ROC) curves were used to identify “optimal” (at least 0.7 for both sensitivity and specificity) cutoff threshold scores on both the AQ and RAADS‐R for identifying autism cases (as measured by an ADOS Module 4 score of 10+). For both questionnaires, sensitivity and specificity, with 95% confidence intervals (CIs) were calculated. For the questionnaires to have opt in potential, specificity needed to be 0.7 or above. Thus, optimal sensitivity and specificity was set to be the highest sensitivity for a specificity ≥0.7.

Test–retest reliability for the repeat questionnaires was assessed using Bland–Altman plots (Bland & Altman, 1986). The intra‐class coefficient was coded using methods described by Baumgartner and Jackson (1995), assuming random measurement error and no systematic bias between tests.

## 3 RESULTS

### 3.1 Response rate and participant characteristics

Between July 2011 and December 2012, 739 of 1,479 (50%) eligible patients from outpatient/community mental health team, inpatient, and other mental health settings agreed to take part in the study and to complete both the AQ and RAADS‐R questionnaires (Figure Figure 1). The researcher recorded gender and broad age group (estimated if not provided) of nonparticipants. The age and gender of participants and nonparticipants was similar, but there were proportionally more male participants (50% participants vs. 46% nonparticipants), and participants were also marginally older (28% participants vs. 24% nonparticipants were aged 50+ years). As expected, refusals (directly to research team member or informed by the health professional) were more common in outpatient/community mental health team settings than in inpatient units (57% and 30%, respectively).

**Figure Figure 1:**
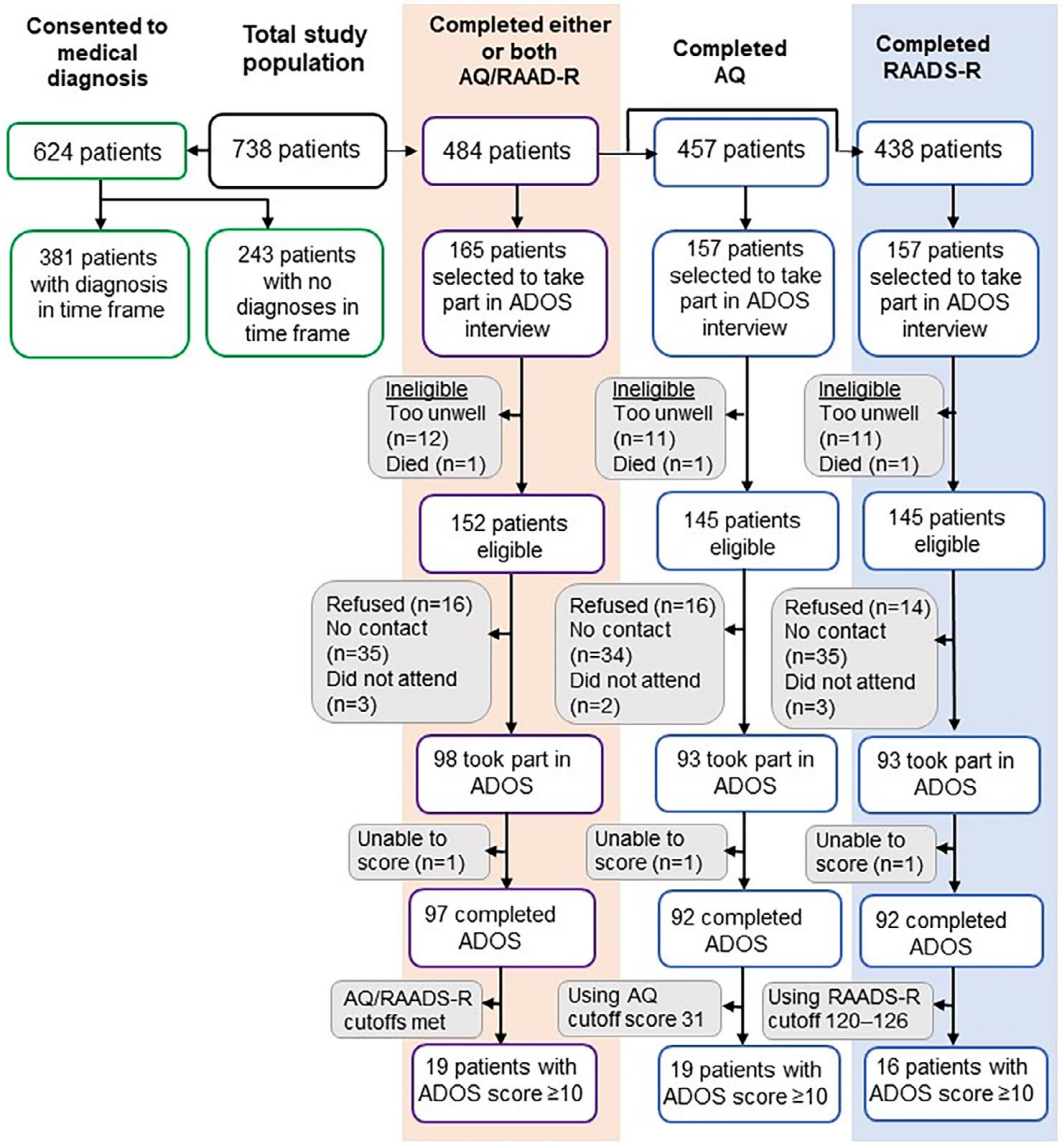
Flow chart demonstrating the multiphase assessment procedure for the study population, with patient numbers. ADOS, autism diagnostic observation schedule; AQ, autism‐spectrum quotient; RAADS‐R, Ritvo autism–Asperger's diagnostic scale‐revised

Of the 738 participants, 457 (62%) completed the AQ and 438 (59%) completed the RAADS‐R. About one‐third of the questionnaires (*n* = 144 AQ and *n* = 172 RAADS‐R) had between one and three missing items requiring imputation. The remaining 254 (34%) participants did not return or failed to fully complete the questionnaires (Table 2). A total of 624 participants allowed access to their recorded psychiatric diagnoses. The most common diagnosis was mood disorder, primarily bipolar affective disorder (*n* = 39; 23% of mood disorders) or depression (*n* = 62; 37% of mood disorders; Table 3).

**Table 2:** Characteristics of participating mental health service users and questionnaire completion by mental health setting

		All settings		Inpatient units		Outpatient/ community mental health settings		Other	
Service users		*N*	(%)	*N*	(%)	*N*	(%)	*N*	(%)
Participants		738	(100.0)	239	(100.0)	476	(100.0)	23	(100.0)
Age group (years)	<40	312	(42.3)	117	(49.0)	189	(39.7)	6	(26.1)
	40–49	220	(29.8)	62	(25.9)	152	(31.9)	6	(26.1)
	50+	206	(27.9)	60	(25.1)	135	(28.4)	11	(47.8)
Gender	Male	366	(49.6)	135	(56.5)	217	(46.6)	14	(60.9)
	Female	372	(50.4)	104	(43.5)	259	(54.4)	9	(39.1)
Questionnaire completion									
AQ		457		185		256		16	
RAADS‐R		438		174		248		16	
Both incomplete		45	(6.1)	17	(7.1)	21	(4.4)	7	(30.4)
Not returned		209	(28.3)	25	(10.5)	184	(38.7)	0	(0.0)

**Table 3:** Mental health service recorded psychiatric diagnoses (by broad ICD‐10 category) of participating mental health service users

Characteristic	Number (%)
Consenting participants^a^	624 (100.0)
No diagnosis within time frame	243 (38.9)
Diagnosis within time frame (ICD‐10)^b^	381 (61.1)
Mental and behavioural disorders due to alcohol/substance misuse (F1x.x)	73 (11.7)
Schizophrenia, schizotypal and delusional disorder (F2x.x)	64 (10.2)
Mood disorder (F3x.x)	167 (26.8)
Neurotic, stress‐related and somatoform disorders (F4x.x)	43 (6.9)
Personality disorder (F6x.x)	61 (9.8)
Autism spectrum disorders (F84.x)	6 (1.0)
Attention deficit hyperactivity disorder (F90.x)	5 (0.8)
Other mental health disorders (F0x.x, F5x.x, F81.x, F95.x)	12 (1.9)

*Note*. Percentages do not always add up to 100.0 because of rounding. Abbreviation: ICD; International Classification of Diseases.

a Number who gave permission to access diagnoses.

b Diagnoses total to 431 owing to individuals having more than one diagnosis.

### 3.2 Internal consistency of AQ and RAADS‐R items

The median total score on the AQ was 22 (range 3 to 46). The scores appeared to be normally distributed (Shapiro–Wilk test *p* = .11; Table 4). Internal consistency for items on the AQ using Cronbach's alpha was good (*α* = 0.85) with the average inter‐item correlation lower than the recommended range (0.15 to 0.50; Simms & Watson, 2007), from 0.097 for Item 47 to 0.106 for Item 9.

**Table 4:** Summary of criterion‐related validity and test–retest reliability findings for the autism‐spectrum quotient and Ritvo autism–Asperger's diagnostic scale‐revised

Content	AQ	RAADS‐R
Distribution of scores	Normally distributed	Not normally distributed
Internal consistency of items	Good internal consistency (*α* = 0.85) Inter‐item correlations outside recommended range	Excellent internal consistency (*α* = 0.95) Inter‐item correlations within recommended range
Diagnostic accuracy	Fair diagnostic accuracy (area under ROC curve 0.77)	Fair diagnostic accuracy (area under ROC curve 0.79)
Sensitivity and specificity	0.79 and 0.77 at cutoff ≥31	0.75 and 0.71 at cutoff ≥120
Test–retest reliability	Excellent (ICC = 0.90)	Excellent (ICC = .88)

Abbreviations: AQ, autism‐spectrum quotient; ICC, intra‐class correlation coefficient; RAADS‐R, Ritvo autism–Asperger's diagnostic scale‐revised; ROC, receiver operating characteristic.

The median total score on the RAADS‐R was 84.5 (range 0 to 207). We found evidence that the scores did not follow a normal distribution (Shapiro–Wilk *p* < .001). Internal consistency for items on the RAADS‐R using Cronbach's alpha was good (*α* = 0.95) with the average inter‐item correlation within the recommended range (.15 to .50; Simms & Watson, 2007), from .202 for Item 56 and Item 60 to .208 for Item 40.

### 3.3 Criterion‐related validity of the AQ and RAADS‐R

One hundred and fifty‐three eligible participants who had completed the AQ and/or the RAADS‐R took part in the reference interview assessment with the ADOS Module 4. In total, 98 interviews were conducted among participants who completed either or both questionnaires. One hundred and forty‐five participants had completed the AQ of whom 93 participants agreed to be interviewed (response rate 64.1%), usually in their own home. One participant had such complex difficulties that the interviewers did not feel confident in scoring the ADOS Module 4. This participant was excluded from the analyses, leaving 92 remaining.

Scores in the AQ and ADOS Module 4 were moderately correlated (Spearman's rho = 0.44) and appeared to follow a normal distribution (Shapiro–Wilk *p* = .11). Scores had good internal consistency (*α* = 0.85), but average inter‐item correlations were lower than the recommended range (.15 to .50; Simms & Watson, 2007) ranging from .097 for Item 47 to .106 for Item 9.

Using the recommended cutoff threshold in the ADOS Module 4 of 7+ for ASD, with a minimum of 2+ for communication and 4+ for reciprocal social interaction, 25 cases of ASD were identified. Nineteen adults met the higher threshold of 10+ for autism (4.8%; 95% CI [2.9, 7.5]): 8 were men and 11 were women.

Of these 19 research‐identified adult autism cases, only one had a recorded service coded diagnosis of ASD prior to entering the study. The most common service diagnoses in those remaining were personality disorders (*n* = 5; 28%) and mood disorders (*n* = 3; 17%).

Using the higher cutoff threshold for autism, the ROC curve for AQ revealed a “fair” diagnostic accuracy (Cicchetti & Sparrow, 1981, Fleiss, 1981; area under the ROC curve = 0.77; Figure Figure 2). Optimal sensitivity and specificity was at a cutoff of 31, with sensitivity 0.79 (95% CI [0.54, 0.94]) and specificity 0.77 (95% CI [0.65, 0.86]).

**Figure Figure 2:**
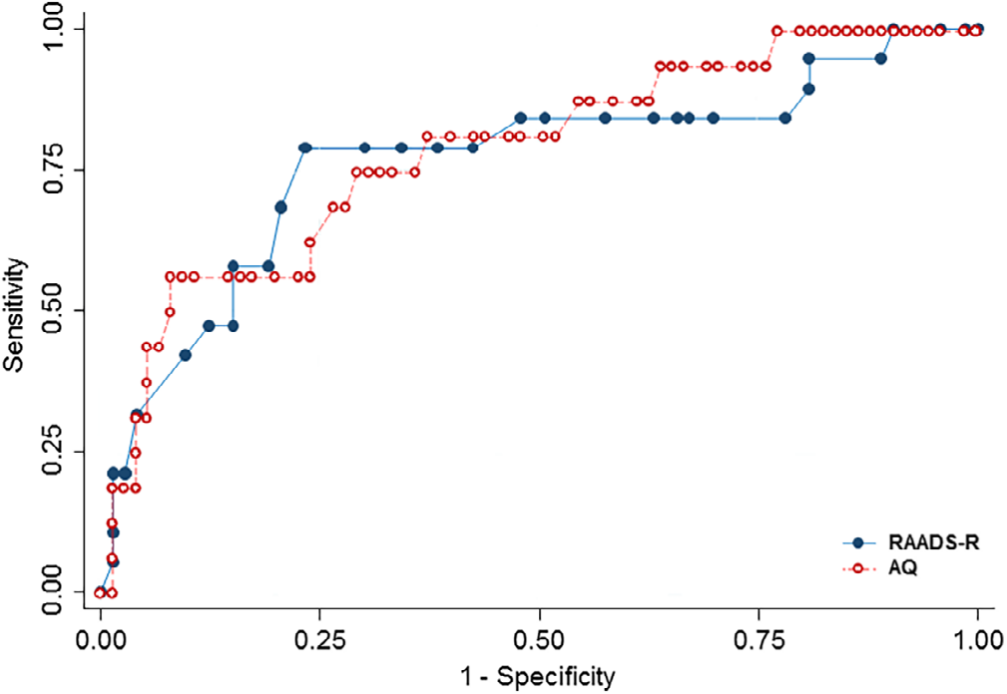
Receiver operating characteristic curve of the autism‐spectrum quotient (AQ) and the Ritvo autism–Asperger's diagnostic scale‐revised (RAADS‐R) against the reference standard autism diagnostic observation schedule Module 4 among participating mental health service users. Area under the ROC curve: AQ 0.77; RAADS‐R 0.79

For adults who had completed the RAADS‐R, the response rate was again 64.1%. As before, one participant was excluded owing to her complex difficulties. Scores in the RAADS‐R and ADOS Module 4 were moderately correlated (Spearman's rho = 0.47), and we found evidence that they did not follow a normal distribution (Shapiro–Wilk *p* < .001). Internal consistency for items was excellent (*α* = 0.95) and average inter‐item correlations were within the recommended range (.15 to .50; Simms & Watson, 2007), ranging from .202 for Item 56 and Item 60 to .208 for Item 40.

Sixteen adults met the higher ADOS threshold of 10+ for autism: 6 were men and 10 were women. As before, the most common service recorded diagnosis, among those remaining research identified adult autism cases, were personality disorders (*n* = 5; 33%) and mood disorders (*n* = 3; 20%).

Using the higher ADOS threshold for autism, the ROC curve for RAADS‐R revealed a “fair” diagnostic accuracy (Cicchetti & Sparrow, 1981, Fleiss, 1981; area under the ROC curve = 0.78; Figure Figure 2). Optimal sensitivity and specificity was at a cutoff of 120–126, with sensitivity 0.75 (95% CI [0.48, 0.93]) and specificity 0.71 (95% CI [0.60, 0.81]).

### 3.4 Test–retest reliability of the AQ and RAADS‐R

Twenty participants completed the AQ, and 26 participants completed the RAADS‐R for a second time within 1–3 months of first completion, with mean baseline AQ scores 21 (range 6–39) and mean baseline RAADS‐R scores 87 (range 6–192). Using Bland–Altman plots (Figure Figure 3,b), we found no significant differences between the mean values for the two administrations of either the AQ or RAADS‐R and no evidence of systematic bias (AQ: mean difference = −0.80; 95% CI [−2.83, 1.23]; *p* = .42, and RAADS‐R: mean difference = −5.19; 95% CI [−16.89, 6.51]; *p* = .37). The intra‐class correlation coefficient for the two AQ tests was .90 (95% CI [0.82, 0.99]) and for the two RAADS‐R tests was .88 (95% CI [0.80, 0.97]) suggesting excellent test–retest reliability (Table 4).

**Figure Figure 3:**
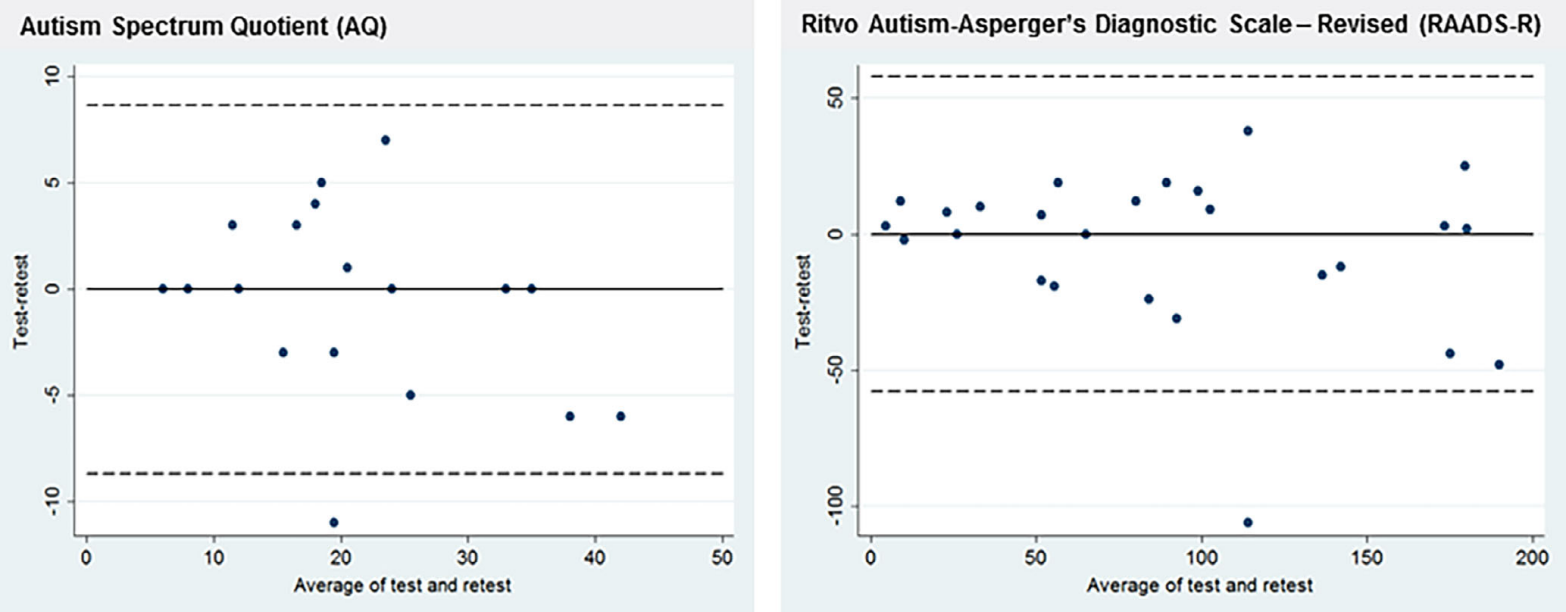
(a) Bland–Altman Plot for test–retest agreement of the Autism Spectrum Quotient (AQ) and (b) Ritvo autism–Asperger's diagnostic scale‐revised (RAADS‐R). Intra‐class correlation coefficient = .90 for AQ; intra‐class correlation coefficient = .88 for RAADS‐R

## 4 DISCUSSION

Overall, the findings suggest that both the self‐completion questionnaires, the AQ and the RAADS‐R, may be useful in facilitating psychiatrists' and psychologists' referrals to ASD services for a diagnostic assessment. However, the questionnaires cannot be used in isolation, but in conjunction with clinical judgement and other ASD assessment tools if necessary. Of note, 6% (*n* = 44) of the participants could not complete either questionnaire, suggesting that a significant minority of mental health services users with capacity struggle with these self‐completion questionnaires and need to be assessed differently. This has implications for their use in clinical practice.

Neither questionnaire appeared to be superior in this study. Items in the AQ were less internally consistent than items in the RAADS‐R, but the AQ appeared to be easier to complete, and we noted that more participants failed to complete the RAADS‐R than the AQ (12% vs. 10%). The optimal threshold for the RAADS‐R was also substantially higher than that recommended by the developers (120 vs. 65), which might, in part, be explained by the different study populations, but merits further investigation. More than half of our service users met the threshold for autism using this recommended (lower) cut off, which is concerning. Our study interviewers remarked that participants seemed to struggle more with the scoring options for RAADS‐R; also they appeared to become confused by what some of the questions were asking, especially those looking at sensory issues or emotions. The RAADS‐R developers encourage clinician support to patients when completing the test but do permit unsupervised use, which would reduce cost of use. In our study, although support was offered to all patients, a minority of patients would insist on taking the questionnaires away with them to complete on their own, potentially impacting on the effectiveness of this measure for such individuals.

Although the male to female ratio was similar among the 392 individuals who completed the questionnaires (48.7% men and 51.3% women), we identified more autism cases among women than men, which is contrary to the literature in the general population where autism is more prevalent among men (Brugha et al., 2011, Fombonne, 2005, Newschaffer et al, 2007). Five of the women with ADOS‐determined autism (45%) had a diagnosis of borderline personality disorder, which might support previous research that ASD can be “masked” by such disorders (Rydén et al., 2008). Alternatively women with undiagnosed ASD appear to be more likely than men to mimic “normal” behaviour and repress autistic behaviour, which can be exhausting and potentially harmful to their mental health (Yaull‐Smith, 2008). However, our findings might also suggest false positives with the ADOS Module 4. We also cannot rule out the possibility that adults with ASD were more likely to agree to take part in the project because they were interested in the subject area.

The high proportion (38.9%) of consenting participants lacking a pre‐existing mental health diagnosis is likely to be because these patients had yet to commence or were still undergoing an initial assessment of their mental health at the time their research assessment was completed, such that a formal diagnosis had not yet been determined and coded (and we did not make a record of delayed codings). Additionally, the approach towards coding for pre‐existing mental health diagnoses being limited to diagnoses within 2 years prior to questionnaire completion (or 5 years in the case of ASD and attention deficit hyperactivity disorder) could miss diagnoses made prior to this point.

Both the AQ and RAADS‐R were evaluated by their original developers using a case–control design that is not recommended in test evaluations (Leeflang, Deeks, Takwoingi, & Macaskill, 2013) and which is likely to result in possibly as much as a threefold overestimation of sensitivity and specificity (Lijmer et al., 1999). This design does not take account of the different prevalence of ASD both in the population in which the questionnaires would be completed and in the normal (neurotypical) control groups. Such tests should be evaluated in samples representative of people whose reference diagnosis is unknown and for whom the test is therefore likely to be used, whereas in both of the developer evaluations, subjects were chosen whose diagnosis was already known. Furthermore, we found that the threshold on the AQ recommended by the original developers performed optimally in our ROC analyses in our methodologically recommended cohort design, which was marked in contrast to the RAADS‐R in which the developers' recommended cutoff threshold that is clearly far too low and should not be used. Therefore, it could be argued that the marked difference in optimal cut point on the RAADS‐R not found in the AQ is due to other population differences between all three of these studies rather than to the case–control design used by both developers, such as differences in population composition and in the underlying disorder prevalence. If so, the regular use of such tests must be verified in local calibration estimations, and the so called recommended thresholds should not be relied upon until evaluated independently. When tested in a randomly selected general population community sample, a reduced version of the AQ performed poorly (Brugha et al., 2011). Therefore, it is somewhat encouraging that in a sample of users of adult mental health services, likely to have high levels of psychiatric comorbidity, it appears to be cost effective (as does the RAADS‐R).

There have been a number of other published independent evaluations of the AQ and the RAADS‐R described in systematic reviews of the literature (National Collaborating Centre for Women's and Children's Health, 2011; National Institute for Health and Care Excellence, 2014) and most recently by Wigham et al. (2018). The sensitivity and specificity of the AQ‐50 and the AQ‐10 were found to be good (≥80%) when comparing archival clinical data from adults with ASD, against a general population group (Wigham et al., 2018). In possibly the only study to evaluate the AQ in a large cohort design (Ashwood et al., 2016), findings were reported using the subject and informant versions of the AQ‐50 and the AQ‐10. Participants were consecutively referred to an ASD assessment clinic, from primary care and from tertiary care settings, and had high rates of comorbid mental health conditions. Across both AQ versions, sensitivity was above 71%, but specificity was less than 38%. The review authors concluded that the findings from these studies suggest that due to low levels of specificity, the AQ is not a reliable indicator of which people should progress to a full ASD assessment (Wigham et al., 2018). The same review (Wigham et al., 2018) covered the few published evaluations of the RAADS‐R drawing the same conclusion that due to poor specificity, it could not be recommended; it is notable that all the evaluations identified used the case–control design. Therefore, the present study appears to be the first to use a cohort design to evaluate the RAADS‐R.

## 5 STRENGTHS AND LIMITATIONS

A major strength of this evaluation is the use of a representative sample of one population of clinical relevance; many such evaluations compare different populations (e.g., people known to have a disease who are compared with controls who are highly unlikely to have the disease) leading to biased overestimation of the sensitivity and specificity of a test (Lijmer et al., 1999). It is an approach that fits with the reason for the choice of a test, which is to assist in identifying individuals from the same population who warrant closer and costlier investigation.

Inevitably, there are some possible study limitations. The findings are dependent on the accuracy of the reference standard measure, the ADOS Module 4, which is a well‐established and validated assessment tool for ASD in the adult general population (Brugha et al., 2012). Our own experience suggests that participants may have scored artificially high in the assessment because of their ongoing mental health difficulties. In total, 392 participants completed either test questionnaire and agreed to take part in a subsequent interview. Assuming that we identified all cases of autism using the weighted sampling strategy for second interview, the 19 cases of autism we identified equates to 4.8% of the mental health service user population, higher than previous estimates of 3% (Nylander & Gillberg, 2001). Furthermore, the finding that only one of the 19 cases of autism identified in the present research was already recognised by the specialist mental health service they were attending is retrospective and does not take account of the fact that some participants had no previous contact with the service or that autism may have been recognised by the service after the participant joined the study. Therefore, recognition levels by services may not be as poor as this finding implies. Additionally, our prevalence estimate and standard error did not take account of the weighted sampling strategy because the study was small and may not be representative of other such population settings. A further limitation is that we did not use a standardised developmental assessment, which is often unobtainable in adulthood, but which is a recommended part of an autism assessment (American Psychiatric Association, 2013), but relied only on one measure (the ADOS) to classify autism cases. Although best practice diagnostic procedures often recommend including the ADOS Module 4 in conjunction with other assessment tools, such as a developmental assessment (Pugliese et al., 2015), our review of the literature on possible limitations of the ADOS show that there has as yet been no definitive research addressing this question. Nevertheless, it cannot be ruled out that the presence of psychiatric comorbidity could have elevated the ADOS scores, which potentially inflated the rate of autism and rates of comorbidity described in this study population, and that an unknown proportion of such cases could have had an onset of social communication psychopathology later than childhood.

The use of a two‐phase survey design is a further limitation; this was necessary as the condition being studied is relatively uncommon and it would have been very costly to conduct ADOS examinations on everyone in the first phase sample, who had completed at least one of the two test questionnaires. Therefore, unfortunately, positive and negative predictive value could not be accurately calculated because both are highly sensitive to the correct classification of cases and non‐cases, which is reduced by the further sampling required in a two phase design. Negative predictive value would have been of value to clinicians as it can be used to underpin a decision that suspicion of a condition is not warranted and further testing and investigation are unnecessary. Indeed, to clinicians, this is a particularly valued property of a test. Further work in this area is clearly required.

Our two test questionnaire results are based on the optimal cut point on each test determined by our ROC analysis. That cut point happens to be identical to that originally recommended by the developers of the AQ, thus satisfying an important prerequisite of test evaluations that the test result is not determined by the criterion measure (i.e., ADOS). However, as pointed out earlier, the RAADS‐R did not satisfy this prerequisite; the cut point recommended by its developers was 65 and not 120 in our evaluation. Therefore, this new higher cut point does need to be evaluated independently.

Strictly speaking, as noted above, a test should be evaluated in the population in which it is likely to be used; therefore, we do not know how these tests would perform in adult mental health patients clinically judged to need testing. Instead, we evaluated these two tests in the whole of a population in which practice until now has rarely been to consider the value of testing for autism. As autism awareness grows and as clinicians and Multi‐disciplinary team (MDTs) are increasingly supported and trained to be more aware of the presence of comorbid autism, the design of such studies should focus instead on those clinically judged to be more likely benefit from such a test. But an advantage of our study is that we have obtained an estimate of the prevalence of autism in the adult mental health service user population and an estimate of its under recognition, neither of which would have been possible if we focused on studying only those likely to be tested in practice.

A final limitation for some users of our findings will be the fact that service users in contact with eating disorder services could not be included in this study.

## 6 CONCLUSIONS AND CLINICAL IMPLICATIONS

The AQ and RAADS‐R can be used to facilitate referrals from mental health settings to autism diagnostic services, but not on their own to determine a diagnosis. But in order to develop the capacity to meet this largely unrecognised need, mental health services will have to adapt. Only one of the 19 cases of autism identified in the present research was already recognised by the specialist mental health service they were attending. This and the finding that almost one in 20 service users has autism suggests that adult psychiatrists and allied mental health professionals should all be trained and experienced in identifying autism.

## CONFLICT OF INTEREST

The authors have no conflict of interest to declare.
